# Supplementary figures and images for: Whole-genome sequencing reveals a novel Renibacterium salmoninarum lineage and suggests geographic endemism combined with anthropogenic spread in the North-East Atlantic Area
Source: Appl Environ Microbiol. 2026 May 21;92(6):e00347-26. doi: 10.1128/aem.00347-26 (PMC13274415; doi:10.1128/aem.00347-26)

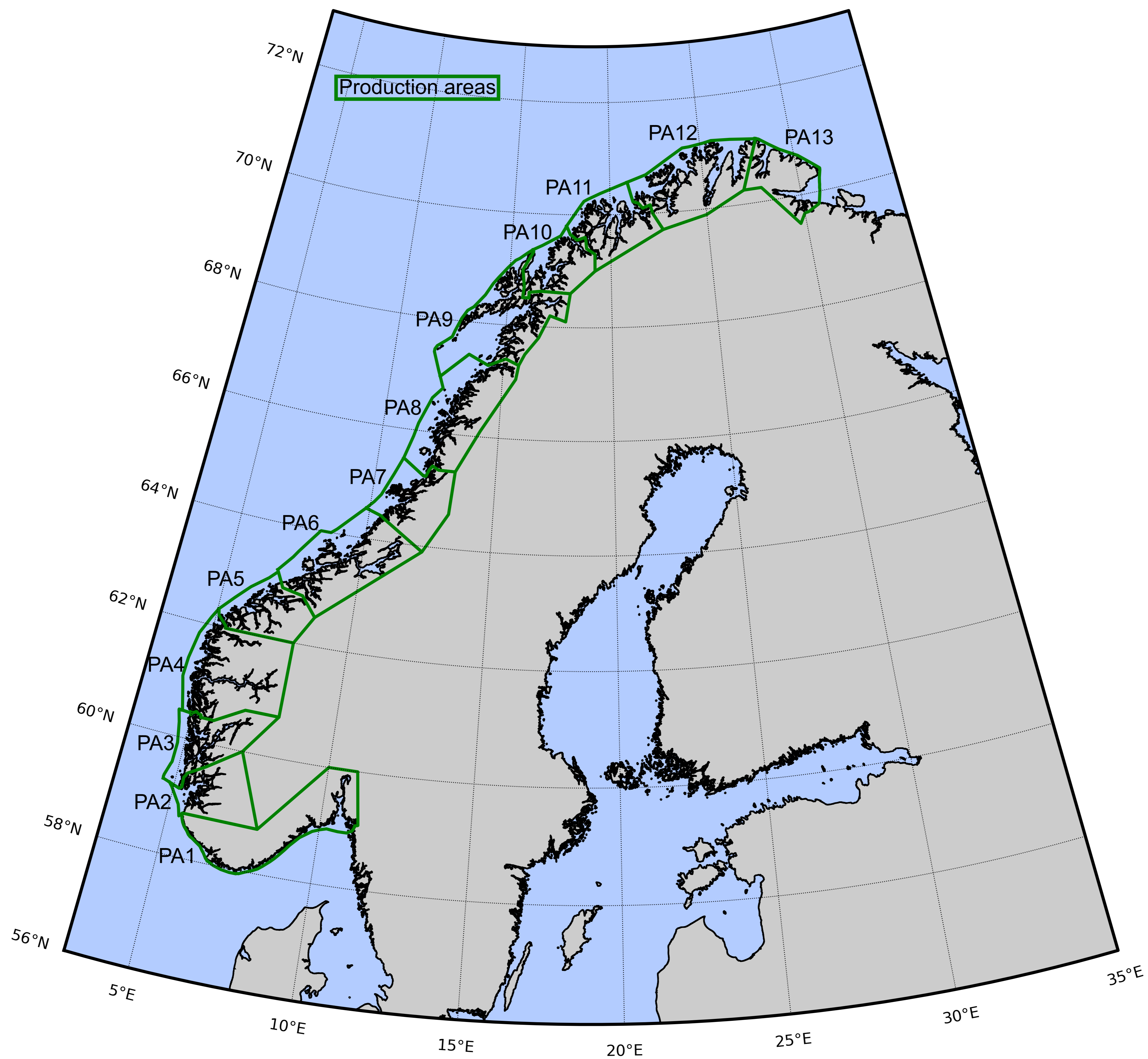

Supplement: Fig. S1 — Production zones 1-13 along the Norwegian coast. [file aem.00347-26-s0001.pdf]

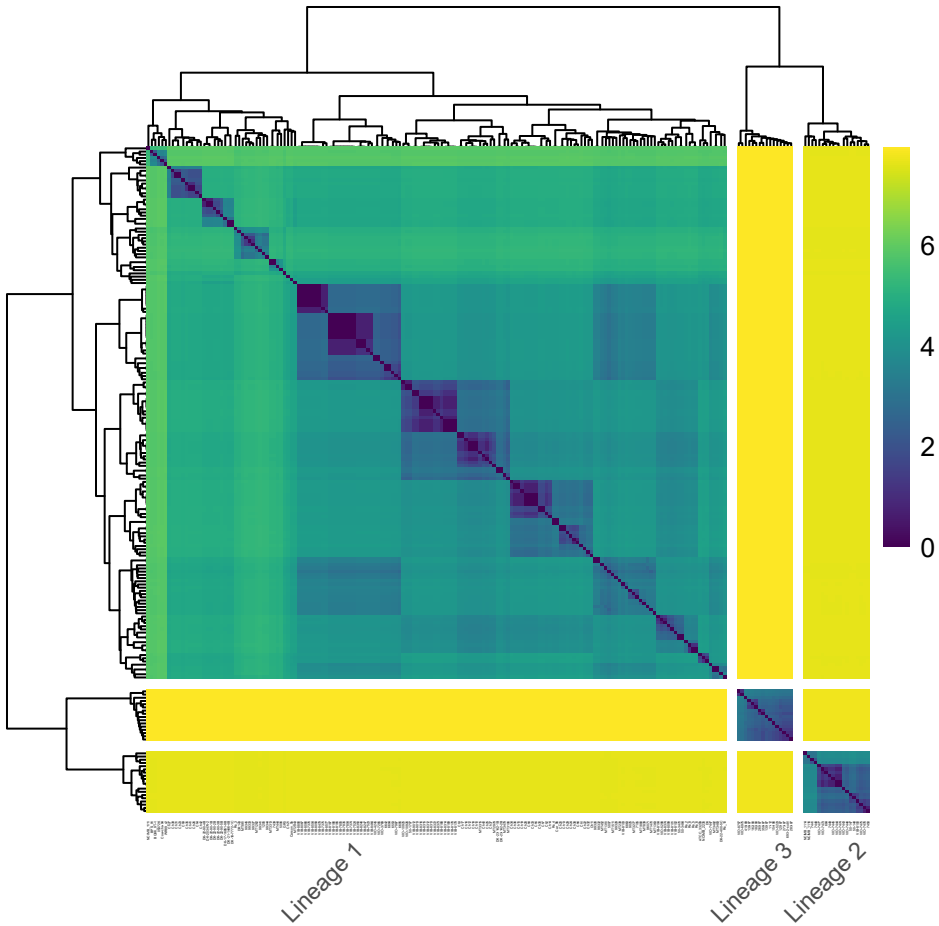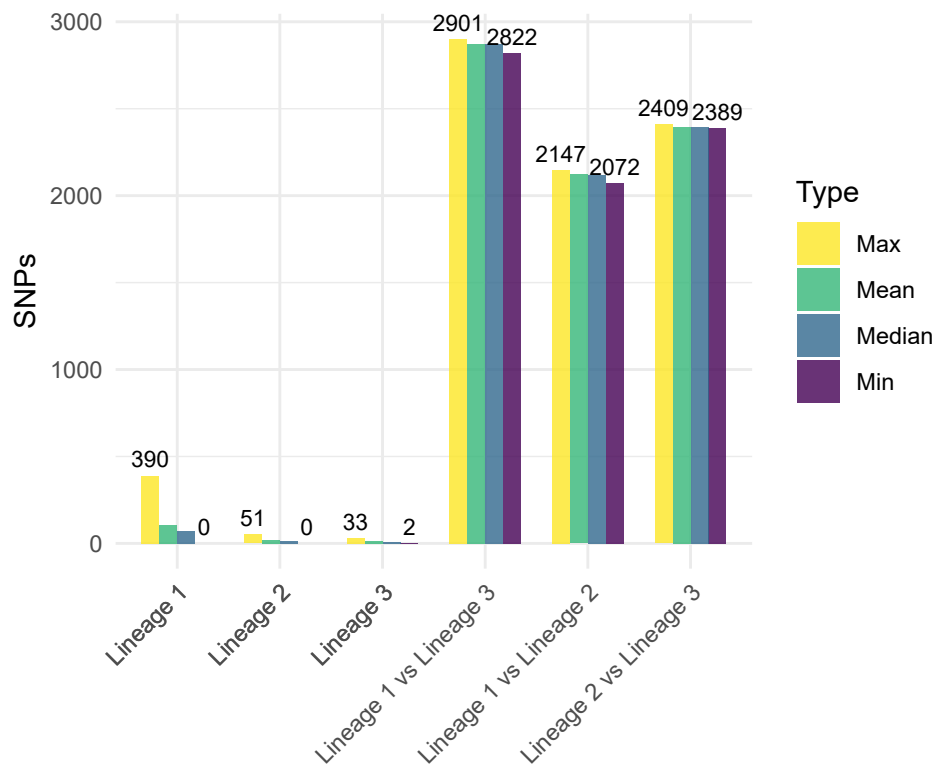

Supplement: Fig. S2 — Heatmap and statistical summary for single nucleotide polymorphism distance matrix for a global collection of 201 Renibacterium salmoninarum genomes. [file aem.00347-26-s0002.pdf]
