# Supplementary material for: Whole-genome sequencing reveals a novel Renibacterium salmoninarum lineage and suggests geographic endemism combined with anthropogenic spread in the North-East Atlantic Area
Source: Appl Environ Microbiol. 2026 May 21;92(6):e00347-26. doi: 10.1128/aem.00347-26 (PMC13274415; doi:10.1128/aem.00347-26)
Supplement: Supplemental legends — Descriptive legends for all supplemental material. [file aem.00347-26-s0003.docx]

Supplementary Figure 1. Production zones 1-13 along the Norwegian coast modified from Lovdata, 2017.

Supplementary Figure 2. Left: Heatmap displaying the single nucleotide polymorphism (SNP) distance matrix for a global collection of 201 Renibacterium salmoninarum genomes. Values were log-transformed, and hierarchical clustering was applied for visualization purposes. Right: Minimum and maximum SNP differences. Yellow, green, blue, and purple bars represent the maximum, median, mean, and minimum SNP differences between isolates within lineages and between lineages.

Supplementary Table 1. Metadata of the 201 Renibacterium salmoninarum isolates analyzed in the study.

Supplementary Table 2. Quality metrics information for the 201 Renibacterium salmoninarum genome assemblies.
