# Supplementary material for: Whole-genome sequencing reveals a novel Renibacterium salmoninarum lineage and suggests geographic endemism combined with anthropogenic spread in the North-East Atlantic Area
Source: Appl Environ Microbiol. 2026 May 21;92(6):e00347-26. doi: 10.1128/aem.00347-26 (PMC13274415; doi:10.1128/aem.00347-26)
Supplement: Table S1 — Metadata of the 201 Renibacterium salmoninarum isolates analyzed in the study. [file aem.00347-26-s0004.pdf]

Supplementary Table 1. Metadata of the 201 *Renibacterium salmoninarum* isolates analyzed in the study.

Isolate	Year	Host species	Country	Production zone (Norway)	Accession number
2005-50	2005	*S. salar*	Norway	-	ERS29020236
50-147	2012	*S. salar*	Norway	4	ERS29020246
50-148	2012	*S. salar*	Norway	4	ERS29020247
50-1707	2016	*S. salar*	Norway	4	ERS29020248
50-3470	2019	*O. mykiss*	Norway	3	ERS29020249
50-3484	2019	*O. mykiss*	Norway	3	ERS29020250
VIB-7374	2023	*S. salar*	Norway	6	ERS29020266
VIB-7375	2023	*S. salar*	Norway	6	ERS29020268
VIB-7376	2023	*S. salar*	Norway	6	ERS29020270
VIB-7523	2023	*S. salar*	Norway	6	ERS29020271
VIB-7524	2023	*S. salar*	Norway	6	ERS29020272
VIB-7804	2023	*S. salar*	Norway	6	ERS29020273
VIB-7805	2023	*S. salar*	Norway	6	ERS29020274
VIB-7835	2023	*S. salar*	Norway	6	ERS29020275
VIB-8058	2023	*S. salar*	Norway	6	ERS29020276
VIB-8066	2023	*S. salar*	Norway	6	ERS29020278
VIB-8067	2023	*S. salar*	Norway	6	ERS29020279
VIB-8099	1998	*S. salar*	Norway	3	ERS29020280

Supplementary Table 1. Metadata of the 201 *Renibacterium salmoninarum* isolates analyzed in the study.

Isolate	Year	Host species	Country	Production zone (Norway)	Accession number
VIB-8100	1998	*S. salar*	Norway	3	ERS29020281
VIB-8101	1999	*S. salar*	Norway	3	ERS29020282
VIB-8102	2003	*S. salar*	Norway	4	ERS29020283
VIB-8103	2001	*S. salar*	Norway	3	ERS29020284
VIB-8104	2005	*S. salar*	Norway	4	ERS29020285
VIB-8178	2023	*O. mykiss*	Norway	4	ERS29020286
VIB-8179	2023	*O. mykiss*	Norway	4	ERS29020287
VIB-8185	2023	*S. salar*	Norway	6	ERS29020288
VIB-8186	2023	*S. salar*	Norway	6	ERS29020289
VIB-8187	2023	*S. salar*	Norway	6	ERS29020290
VIB-8188	2023	*O. mykiss*	Norway	4	ERS29020291
VIB-8189	2023	*O. mykiss*	Norway	4	ERS29020292
VIB-8246	2023	*S. salar*	Norway	6	ERS29020293
VIB-8247	2023	*S. salar*	Norway	6	ERS29020294
VIB-8312	2023	*S. salar*	Norway	4	ERS29020295
VIB-8313	2023	*S. salar*	Norway	4	ERS29020296
VIB-8314	2023	*S. salar*	Norway	4	ERS29020297
VIB-8315	2023	*S. salar*	Norway	4	ERS29020298

Supplementary Table 1. Metadata of the 201 *Renibacterium salmoninarum* isolates analyzed in the study.

Isolate	Year	Host species	Country	Production zone (Norway)	Accession number
VIB-8316	2023	*S. salar*	Norway	4	ERS29020299
VIB-8350	2023	*S. salar*	Norway	6	ERS29020300
VIB-8372	2023	*S. salar*	Norway	4	ERS29020301
VIB-8373	2023	*S. salar*	Norway	4	ERS29020302
VIB-8374	2005	*S. salar*	Norway	4	ERS29020303
VIB-8464	2023	*S. salar*	Norway	6	ERS29020237
VIB-8479	2023	*S. salar*	Norway	6	ERS29020238
VIB-8504	2024	*S. salar*	Norway	6	ERS29020240
VIB-8577	2024	*S. salar*	Norway	6	ERS29020239
VIB-8890	2024	*S. salar*	Norway	6	ERS29020304
VIB-9012	2024	*S. salar*	Norway	5	ERS29020264
VIB-9013	2024	*S. salar*	Norway	5	ERS29020265
VIO-10925	2017	*S. salar*	Norway	4	ERS29020305
VIO-10926	2017	*S. salar*	Norway	4	ERS29020306
VIO-3769	1997	*S. salar (wf)*	Norway	6	ERS29020307
VIO-4245	2000	*S. salar (wf)*	Norway	6	ERS29020308
VIO-5223	2005	*S. salar*	Norway	3	ERS29020309
VIO-5223A	2005	*S. salar*	Norway	-	ERS29020310

Supplementary Table 1. Metadata of the 201 *Renibacterium salmoninarum* isolates analyzed in the study.

Isolate	Year	Host species	Country	Production zone (Norway)	Accession number
VIO-5223B	2005	*S. salar*	Norway	-	ERS29020311
VIO-5298	2005	*S. salar*	Norway	4	ERS29020312
VIO-6975	2009	*S. salar*	Norway	13	ERS29020313
VIO-7285	2000	*S. salar (wf)*	Norway	6	ERS29020314
VIO-7443	1985	*S. salar*	Norway	3	ERS29020315
VIO-7444	1985	*S. salar*	Norway	8	ERS29020316
VIO-7445	1985	*S. salar*	Norway	8	ERS29020317
VIO-7446	1987	*S. salar (wf)*	Norway	4	ERS29020318
VIO-7447	1987	*O. mykiss*	Norway	4	ERS29020319
VIO-7473	1986	*S. trutta*	Norway	4	ERS29020320
VIO-7474	1987	*S. salar*	Norway	4	ERS29020321
VIO-7475	1987	*S. salar*	Norway	3	ERS29020322
VIO-7476	1987	*S. salar*	Norway	4	ERS29020323
VIO-7477	1987	*S. salar*	Norway	4	ERS29020324
VIO-7987	2011	*S. salar*	Norway	9	ERS29020325
VIO-8757	2012	*S. salar*	Norway	13	ERS29020326
DK-17-16851-9B	2017	*O. mykiss*	Denmark	-	ERS29020252
DK-18-7772-1C	2018	*O. mykiss*	Denmark	-	ERS29020253

Supplementary Table 1. Metadata of the 201 *Renibacterium salmoninarum* isolates analyzed in the study.

Isolate	Year	Host species	Country	Production zone (Norway)	Accession number
DK-203474-2	-	*O. mykiss*	Denmark	-	ERS29020254
DK-21-1446-4B	2021	*O. mykiss*	Denmark	-	ERS29020255
DK-22-400-5C	2023	*O. mykiss*	Denmark	-	ERS29020256
DK-22-90-1B	2022	*O. mykiss*	Denmark	-	ERS29020257
DK-22-90-2B	2022	*O. mykiss*	Denmark	-	ERS29020258
DK-22-90-3B	2022	*O. mykiss*	Denmark	-	ERS29020259
DK-22-90-4B	2022	*O. mykiss*	Denmark	-	ERS29020260
DK-22-90-5B	2022	*O. mykiss*	Denmark	-	ERS29020261
DK-23-142-1B	2023	*O. mykiss*	Denmark	-	ERS29020262
DK-23-142-2B	2023	*O. mykiss*	Denmark	-	ERS29020263
693-2-sub1	1992	*S. salar*	Faroe Islands	-	ERS29020251
11-12	2012	*S. salar*	Iceland	-	ERS29020229
120-87	1987	*S. salar*	Iceland	-	ERS29020230
130-87	1987	*O. mykiss*	Iceland	-	ERS29020231
138-87	1987	*S. salar*	Iceland	-	ERS29020232
154-96	1996	*S. alpinus*	Iceland	-	ERS29020233
162-90	1990	*S. salar*	Iceland	-	ERS29020234
182-90	1990	-	Iceland	-	ERS29020235

Supplementary Table 1. Metadata of the 201 *Renibacterium salmoninarum* isolates analyzed in the study.

Isolate	Year	Host species	Country	Production zone (Norway)	Accession number
260-87	1987	*S. salar*	Iceland	-	ERS29020241
273-87	1987	*S. salar*	Iceland	-	ERS29020242
283-87	1987	*S. salar*	Iceland	-	ERS29020243
358-87	1987	-	Iceland	-	ERS29020244
419-89	1989	*S. salar*	Iceland	-	ERS29020245
6553	2008	*S. salar*	Norway	6	ERR327955
6642	2008	*S. salar*	Norway	6	ERR327956
6694	2008	*O. mykiss*	Norway	6	ERR327962
6695	2008	*O. mykiss*	Norway	6	ERR327968
684	1986	*S. trutta*	Norway	4	ERR327958
6863	2009	*O. mykiss*	Norway	4	ERR327965
7439	1984	*S. salar*	Norway	4	ERR327971
7441	1985	*S. salar*	Norway	5	ERR327966
7448	1986	*S. salar*	Norway	6	ERR327970
7449	1987	*S. salar*	Norway	11	ERR327969
7450	1987	*S. salar*	Norway	3	ERR327967
5006	1996	*O. kisutch*	Canada	-	ERR327942
BPS_91	1991	*O. gorbuscha*	Canada	-	ERR327952

Supplementary Table 1. Metadata of the 201 *Renibacterium salmoninarum* isolates analyzed in the study.

Isolate	Year	Host species	Country	Production zone (Norway)	Accession number
BQ96_91–1	1996	*O. kisutch*	Canada	-	ERR327963
DR143	1972	*S. fontinalis*	Canada	-	ERR327954
Rs_10	2009	*S. salar*	Canada	-	ERR327945
Rs_2	2005	*S. salar*	Canada	-	ERR327951
Rs_3	2005	*S. salar*	Canada	-	ERR327947
Rs_4	2006	*S. salar*	Canada	-	ERR327946
Rs_5	2007	*S. salar*	Canada	-	ERR327950
Rs_6	2007	*S. salar*	Canada	-	ERR327953
Rs_8	2008	*S. salar*	Canada	-	ERR327944
Ch1	2015	*S. salar*	Chile	-	ERR2540469
Ch10	2012	*S. salar*	Chile	-	ERR2540490
Ch11	2012	*S. salar*	Chile	-	ERR2540478
Ch12	2013	*S. salar*	Chile	-	ERR2540492
Ch13	2013	*O. kisutch*	Chile	-	ERR2540499
Ch14	2013	*S. salar*	Chile	-	ERR2540484
Ch15	2015	*S. salar*	Chile	-	ERR2540480
Ch16	2012	*S. salar*	Chile	-	ERR2540487
Ch17	2013	*S. salar*	Chile	-	ERR2540491

Supplementary Table 1. Metadata of the 201 *Renibacterium salmoninarum* isolates analyzed in the study.

Isolate	Year	Host species	Country	Production zone (Norway)	Accession number
Ch18	2013	*S. salar*	Chile	-	ERR2540489
Ch19	2013	*S. salar*	Chile	-	ERR2540481
Ch2	2014	*S. salar*	Chile	-	ERR2540470
Ch20	2016	*S. salar*	Chile	-	ERR2540472
Ch21	2014	*S. salar*	Chile	-	ERR2540494
Ch22	2013	*S. salar*	Chile	-	ERR2540477
Ch23	2015	*O. kisutch*	Chile	-	ERR2540507
Ch24	2015	*S. salar*	Chile	-	ERR2540468
Ch25	2013	*O. kisutch*	Chile	-	ERR2540500
Ch26	2015	*O. kisutch*	Chile	-	ERR2540508
Ch27	2016	*S. salar*	Chile	-	ERR2540493
Ch28	2012	*S. salar*	Chile	-	ERR2540485
Ch29	2013	*O. kisutch*	Chile	-	ERR2540498
Ch3	2013	*S. salar*	Chile	-	ERR2540488
Ch30	2015	*S. salar*	Chile	-	ERR2540467
Ch31	2015	*O. kisutch*	Chile	-	ERR2540506
Ch32	2015	*S. salar*	Chile	-	ERR2540471
Ch33	2012	*S. salar*	Chile	-	ERR2540475

Supplementary Table 1. Metadata of the 201 *Renibacterium salmoninarum* isolates analyzed in the study.

Isolate	Year	Host species	Country	Production zone (Norway)	Accession number
Ch34	2016	*S. salar*	Chile	-	ERR2540474
Ch35	2013	*O. kisutch*	Chile	-	ERR2540504
Ch36	2016	*S. salar*	Chile	-	ERR2540473
Ch37	2013	*O. mykiss*	Chile	-	ERR2540495
Ch38	2016	*S. salar*	Chile	-	ERR2540476
Ch39	2015	*O. kisutch*	Chile	-	ERR2540503
Ch4	2015	*O. kisutch*	Chile	-	ERR2540502
Ch40	2016	*S. salar*	Chile	-	ERR2540486
Ch41	2016	*O. mykiss*	Chile	-	ERR2540496
Ch42	2016	*O. kisutch*	Chile	-	ERR2540505
Ch5	2013	*S. salar*	Chile	-	ERR2540482
Ch6	2013	*O. kisutch*	Chile	-	ERR2540497
Ch7	2015	*S. salar*	Chile	-	ERR2540479
Ch8	2013	*S. salar*	Chile	-	ERR2540483
Ch9	2013	*O. kisutch*	Chile	-	ERR2540501
1205	2001	*O. mykiss*	UK	-	ERR327930
5007	2005	*O. mykiss*	UK	-	ERR327923
7105	2007	*O. mykiss*	UK	-	ERR327932

Supplementary Table 1. Metadata of the 201 *Renibacterium salmoninarum* isolates analyzed in the study.

Isolate	Year	Host species	Country	Production zone (Norway)	Accession number
9025	2009	*O. mykiss*	UK	-	ERR327912
96071	1996	*O. mykiss*	UK	-	ERR327927
99326	1999	*O. mykiss*	UK	-	ERR327938
99327	1997	*O. mykiss*	UK	-	ERR327931
99329	1998	*O. mykiss*	UK	-	ERR327937
99332	1999	*O. mykiss*	UK	-	ERR327943
99333	1998	*O. mykiss*	UK	-	ERR327921
99341	1998	*O. mykiss*	UK	-	ERR327949
99344	1998	*O. mykiss*	UK	-	ERR327940
99345	1998	*O. mykiss*	UK	-	ERR327948
MT1262	1992	*S. salar*	UK	-	ERR327922
MT1351	1993	*S. salar*	UK	-	ERR327904
MT1363	1993	*O. mykiss*	UK	-	ERR327920
MT1470	1994	*O. mykiss*	UK	-	ERR327910
MT1511	1994	*O. mykiss*	UK	-	ERR327914
MT1880	1996	*S. salar*	UK	-	ERR327909
MT239	1988	*S. salar*	UK	-	ERR327913
MT2943	2005	*S. salar*	UK	-	ERR327936

Supplementary Table 1. Metadata of the 201 *Renibacterium salmoninarum* isolates analyzed in the study.

Isolate	Year	Host species	Country	Production zone (Norway)	Accession number
MT2979	2005	*O. mykiss*	UK	-	ERR327935
MT3106	2006	*O. mykiss*	UK	-	ERR327939
MT3277	2008	*O. mykiss*	UK	-	ERR327926
MT3313	2008	*O. mykiss*	UK	-	ERR327925
MT3315	2008	*O. mykiss*	UK	-	ERR327928
MT3479	2008	*S. salar*	UK	-	ERR327933
MT3482	2009	*S. salar*	UK	-	ERR327934
MT3483	2009	*S. salar*	UK	-	ERR327941
MT444	1988	*S. salar*	UK	-	ERR327916
MT452	1988	*O. mykiss*	UK	-	ERR327918
MT839	1990	*S. salar*	UK	-	ERR327917
MT861	1990	*S. salar*	UK	-	ERR327919
NCIMB_1114	1962	*S. salar*	UK	-	ERR327908
NCIMB_1116	1962	*S. salar*	UK	-	ERR327907
05372K	2005	*O. tshawytscha*	USA	-	ERR327906
ATCC_33209	1974	*O. tshawytscha*	USA	-	ATCC33209
Car_96	1996	*O. tshawytscha*	USA	-	ERR327957
Carson_5b	1994	*O. tshawytscha*	USA	-	ERR327905

Supplementary Table 1. Metadata of the 201 *Renibacterium salmoninarum* isolates analyzed in the study.

Isolate	Year	Host species	Country	Production zone (Norway)	Accession number
Cow-chs-94	1994	*O. tshawytscha*	USA	-	ERR327915
D6	1982	*O. tshawytscha*	USA	-	ERR327961
GR5	1997	*T. thymallus*	USA	-	ERR327959
NCIMB_2235	1974	*O. tshawytscha*	USA	-	ERR327911
WR99_c2	1999	*O. kisutch*	USA	-	ERR327960
NCIMB_1111	-	-	USA/UK	-	ERR327924

(wf)= originated from wild fish
